# Prognostic role of the ABO blood types in Chinese patients with curatively resected non-small cell lung cancer: a retrospective analysis of 1601 cases at a single cancer center

**DOI:** 10.1186/s40880-015-0054-2

**Published:** 2015-09-28

**Authors:** Ning Li, Miao Xu, Chao-Feng Li, Wei Ou, Bao-Xiao Wang, Song-Liang Zhang, Peng-Fei Xu, Cheng Yuan, Qun-Ai Huang, Si-Yu Wang

**Affiliations:** Sun Yat-sen University Cancer Center, State Key Laboratory of Oncology in South China, Collaborative Innovation Center for Cancer Medicine, Guangzhou, 510060 Guangdong P.R. China; Department of Thoracic Surgery, Sun Yat-sen University Cancer Center, Guangzhou, 510060 Guangdong P.R. China; Department of Experimental Research, Sun Yat-sen University Cancer Center, Guangzhou, 510060 Guangdong P.R. China; Department of Information Technology, Sun Yat-sen University Cancer Center, Guangzhou, 510060 Guangdong P.R. China; Breast Tumor Center, Sun Yat-sen Memorial Hospital, Guangzhou, 510120 Gaungdong P.R. China; Department of Thyroid and Breast Surgery, The Third Affiliated Hospital of Sun Yat-sen University, Guangzhou, 510630 Guangdong P.R. China

**Keywords:** The ABO blood types, Lung cancer, Prognosis, Survival

## Abstract

**Background:**

A positive association between the ABO blood types and survival has been suggested in several malignancies. The aim of this study was to assess the role of the ABO blood types in predicting the prognosis of Chinese patients with curatively resected non-small cell lung cancer (NSCLC).

**Methods:**

We retrospectively analyzed 1601 consecutive Chinese patients who underwent curative surgery for NSCLC between January 1, 2005 and December 31, 2009. The relationship between the ABO blood types and survival was investigated. In addition, univariate and multivariate analyses were performed.

**Results:**

Group 1 (patients with the blood type O or B) had significantly prolonged overall survival (OS) compared with group 2 (patients with the blood type A or AB), with a median OS of 74.9 months versus 61.5 months [hazard ratio (HR) 0.83; 95% confidence interval (CI) 0.72–0.96; *P* = 0.015]. Additionally, group 1 had significantly longer disease-free survival (DFS; HR 0.86; 95% CI 0.76–0.98; *P* = 0.022) and locoregional relapse-free survival (LRFS; HR 0.79; 95% CI 0.64–0.98; *P* = 0.024) than group 2. The association was not significantly modified by other risk factors for NSCLC, including smoking status, pathologic tumor-node-metastasis stage, pT category, pN category, and chemotherapy.

**Conclusions:**

There is an association between the ABO blood types and the survival of Chinese patients with resected NSCLC. Patients with the blood type O or B had significantly prolonged OS, DFS, and LRFS compared with those with the blood type A or AB.

## Background

Globally, lung cancer remains the most common cancer for both men and women and accounts for 13% of total cases and 18% of total deaths [1]. Lung cancer has also been the top one malignancy in terms of incidence and mortality in China [2, 3]. Non-small cell lung cancer (NSCLC) accounts for 80%–85% of 1.1 million newly diagnosed lung cancer cases annually [4]. Although surgical resection with curative intent was available, 40%–75% of patients died within 5 years [5]. Understanding the etiology of this typically fatal disease and identifying novel prognostic factors are essential for early diagnosis, prognosis evaluation, and more appropriate treatment.

The ABO blood type is determined by terminal carbohydrates expressed on red blood cells, which are attached to a protein backbone, the H antigen. The glycosyltransferase, which is encoded by the ABO gene on chromosome 9q34, can catalyze the transfer of donor sugars to the H antigen to form the ABO blood type antigens [6, 7]. A genome-wide association study (GWAS) of pancreatic cancer identified a genetic variation in the ABO locus of 9q34 that was associated with susceptibility to pancreatic cancer [8, 9]. The positive association between the ABO blood types and survival has been suggested in several malignancies [10], including pancreatic cancer [11, 12], breast cancer [13, 14], renal cell carcinoma [15], nasopharyngeal carcinoma [16], and colon cancer [17]. Although the study by Lee et al. [18] investigated the survival of patients with curatively resected NSCLC within the context of the ABO blood types, the aim of their study was to investigate the prognostic role of the expression of blood group antigen A in tumor cells.

We conducted this study to investigate the relationship between the ABO blood types and the survival of patients who underwent primary curative resection. We also evaluated the associations between the ABO blood types and other clinicopathologic features of NSCLC to determine whether the ABO blood types are independent prognostic factors. In the present paper, we report results from Chinese patients with curatively resected NSCLC.

## Patients and methods

### Patient selection and data collection

A retrospective analysis was performed on consecutive patients who underwent curative surgery for NSCLC at the Sun Yat-sen University Cancer Center between January 1, 2005 and December 31, 2009. This study was approved by the Medical Ethics Committee and Clinical Trial Review Committee of this cancer center. All patients had postoperatively, pathologically confirmed NSCLC without previous therapy other than complete resection and neoadjuvant chemotherapy. The main exclusion criteria included incomplete resection, previous malignant disease, and perioperative death. Informed consent was obtained from all individual participants included in the study.

Data were collected from electronic and paper patient medical records, and survival data were obtained from the cancer center’s follow-up registry. The data collected included age, sex, Eastern Cooperative Oncology Group (ECOG) performance status, smoking status, pathology, tumor-node-metastasis (TNM) stage, dates of surgery and relapse/metastasis, and the ABO blood type. Patients with insufficient data were excluded from this study. All patients were restaged by using the 7th international system for lung cancer staging [19].

### Study endpoints

The following endpoints were estimated: overall survival (OS), defined as the interval from the date of surgery to the date of death from any cause; disease-free survival (DFS), defined as the interval from the date of surgery to the date of disease recurrence or death from any cause; and locoregional relapse-free survival (LRFS) and distant metastasis-free survival (DMFS), defined as the interval from the date of surgery to the date of locoregional relapse and distant metastasis, respectively.

### Statistical analysis

All endpoints were estimated by the Kaplan–Meier method and compared by using the log-rank test. Multivariate analyses were carried out by using the Cox proportional hazards model to identify important prognostic factors for OS. All variables reaching a significance of 0.1 in univariate analyses were tested in the Cox model. Two-sided *P* values of <0.05 were considered statistically significant. All analyses were performed using the SPSS16.0 software (SPSS Inc., Chicago, IL, USA).

## Results

### Patient population

**Fig. 1:**
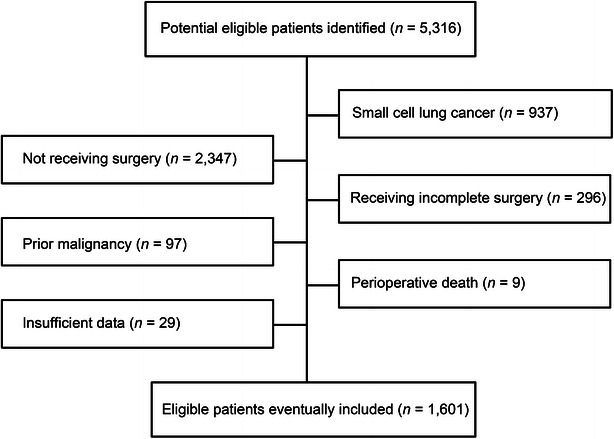
The process of patient selection in this study. A total of 1601 patients with non-small cell lung cancer who underwent curative resection were included in this study

A total of 1601 patients with NSCLC who underwent curative resection were included in this study (Table 1; Fig. 1). In the present study, blood types A, B, O, and AB were reported in 27.7%, 26.5%, 39.2%, and 6.6% of the patients, respectively, which were similar to those frequencies reported previously for the Guangdong population (type A, 25.02%; type B, 25.91%; type O, 42.96%; and type AB, 6.11%) [20]. As shown in Table 1, there were no significant differences in the basic characteristics of our study population based on the blood type. Overall, more than one-half of the patients were presented with adenocarcinoma, and most of the patients were males. Current smokers made up approximately half of all patients.

**Table 1:** Basic characteristics in distinct ABO blood type groups of patients with non-small cell lung cancer (NSCLC)

Characteristic	ABO blood type				*P* value^a^
	O	A	B	AB	
Total (cases)	627	443	425	106	
Age					
Median (years)	59	59	59	59	
Range (years)	30–82	24–80	19–84	23–81	
<55 years [cases (%)]	156 (25.2)	135 (31.8)	207 (33.0)	32 (30.2)	0.11
55–64 years [cases (%)]	137 (30.9)	171 (40.2)	239 (38.1)	43 (40.6)	
>64 years [cases (%)]	150 (33.9)	119 (28.0)	181 (28.9)	31 (29.2)	
Sex [cases (%)]					0.20
Males	451 (71.9)	329 (74.3)	297 (69.9)	81 (76.4)	
Females	176 (28.1)	114 (25.7)	128 (30.1)	25 (23.6)	
ECOG PS score [cases (%)]					0.94
0	305 (48.6)	211 (47.6)	199 (46.8)	52 (49.1)	
1	322 (51.4)	232 (52.4)	226 (53.2)	54 (50.9)	
Smoking status [cases (%)]					0.21
Never	243 (38.8)	177 (40.0)	185 (43.5)	37 (34.9)	
Former	74 (11.8)	59 (13.3)	35 (8.2)	13 (12.3)	
Current	310 (49.4)	207 (46.7)	205 (48.2)	56 (52.8)	
Pathology [cases (%)]					0.67
Squamous cell carcinoma	242 (38.6)	149 (33.6)	142 (33.4)	38 (35.8)	
Adenocarcinoma	327 (52.2)	256 (57.8)	250 (58.8)	58 (54.7)	
Adenosquamous carcinoma	41 (6.5)	24 (5.4)	21 (4.9)	7 (6.6)	
Others	17 (2.8)	14 (3.2)	12 (2.8)	3 (1.8)	
pT category [cases (%)]					0.80
1	112 (17.9)	81 (18.3)	68 (16.0)	17 (16.0)	
2	393 (62.7)	274 (61.9)	288 (67.8)	68 (64.2)	
3	70 (11.2)	50 (11.3)	43 (10.1)	14 (13.2)	
4	52 (8.3)	38 (8.6)	26 (6.1)	7 (6.6)	
pN category [cases (%)]					0.62
0	372 (59.3)	239 (54.0)	232 (54.6)	62 (58.5)	
1	77 (12.3)	60 (13.5)	62 (14.6)	14 (13.2)	
2	178 (28.4)	144 (32.5)	131 (30.8)	30 (28.3)	
pTNM stage [cases (%)]					0.88
Ia	80 (12.8)	58 (13.1)	52 (12.2)	13 (12.3)	
Ib	231 (36.8)	144 (32.5)	155 (36.5)	37 (34.9)	
IIa	18 (2.9)	11 (2.5)	10 (2.4)	1 (0.9)	
IIb	91 (14.5)	62 (14.0)	59 (13.9)	20 (18.9)	
IIIa	207 (33.0)	168 (37.9)	149 (35.0)	35 (33.0)	
Surgery type [cases (%)]					0.97
Lobectomy	506 (80.7)	356 (80.4)	344 (80.9)	87 (82.1)	
Pneumonectomy	96 (15.3)	66 (14.9)	67 (15.8)	15 (14.2)	
Others	25 (4.0)	21 (4.7)	14 (3.3)	4 (3.7)	
Chemotherapy [cases (%)]					0.81
Neoadjuvant + adjuvant	14 (2.2)	13 (2.9)	13 (3.1)	3 (2.8)	
Neoadjuvant only	19 (3.0)	11 (2.5)	11 (2.6)	6 (5.7)	
Adjuvant only	389 (62.0)	287 (64.8)	272 (64.0)	66 (62.3)	
No	205 (32.7)	132 (29.8)	129 (30.4)	31 (29.2)	

*ECOG PS* Eastern Cooperative Oncology Group performance status, *pT category* pathologic tumor category, *pN category* pathologic node category, *pTNM stage* pathologic tumor-node-metastasis stage

^a^χ^2^ test (multigroup comparison)

### Associations between the ABO blood type and survival

**Fig. 2:**
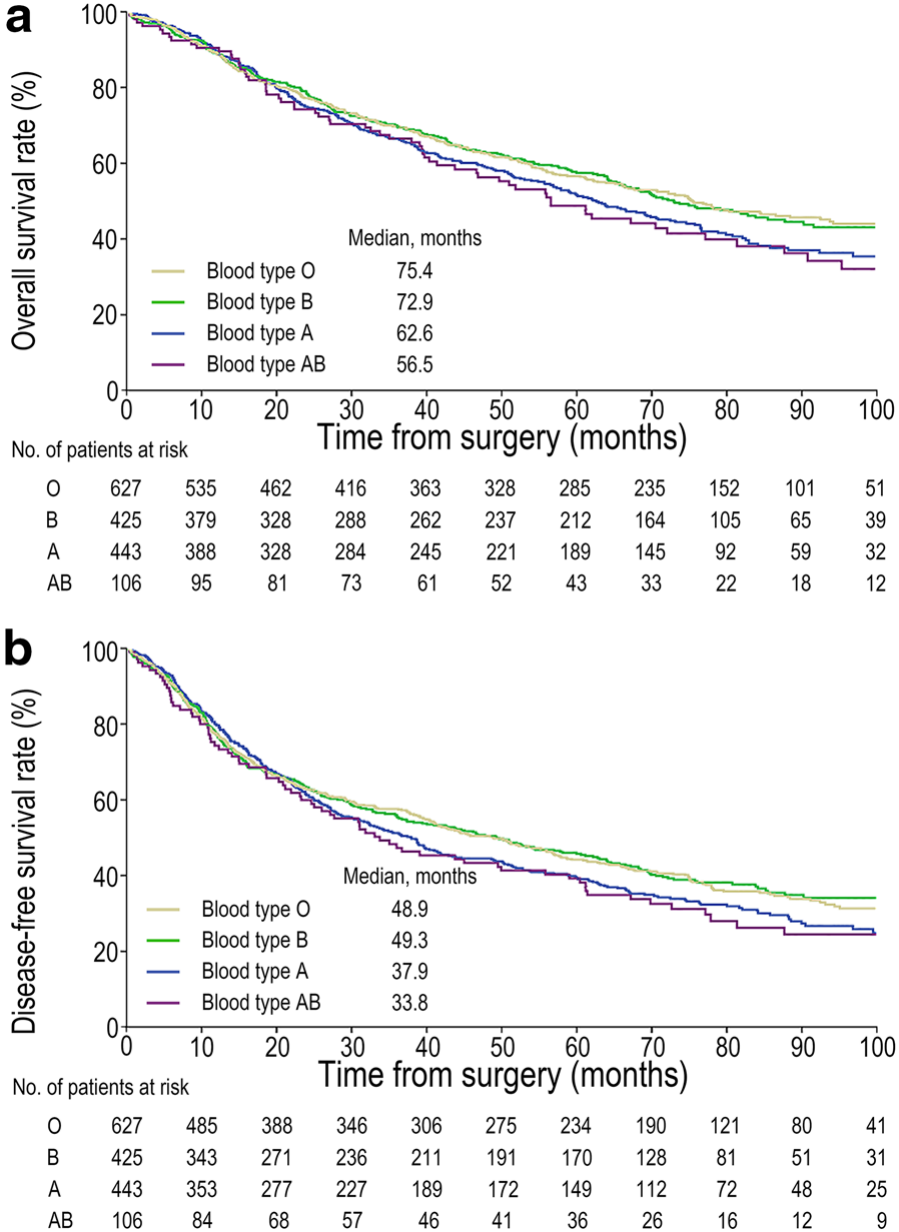
Kaplan–Meier survival curves stratified by the ABO blood type in 1601 patients with curatively resected non–small cell lung cancer. **a** Overall survival for four groups stratified by the ABO blood type. **b** Disease-free survival for four groups stratified by the ABO blood type

The median follow-up was 81.0 months [95% confidence interval (CI) 78.9–83.1 months] for the entire study population. By the time of analysis (January 15, 2015), 360 instances of locoregional relapse, 371 instances of distant metastases, and 810 instances of death had occurred. The median OSs for patients with blood types O, B, A, and AB were 75.4, 72.9, 62.6, and 56.5 months, respectively (*P* = 0.083; Fig. 2a); the median DFSs were 48.9, 49.3, 37.9, and 33.8 months for those with blood types O, B, A, and AB, respectively (*P* = 0.128, Fig. 2b). We found that patients with the blood type O or B had longer OS and DFS compared with those with the blood type A or AB, whereas OS and DFS were similar between patients with the blood types O and B as well as between those with the blood types A and AB. Therefore, we divided the entire cohort into group 1 (patients with the blood type O or B) and group 2 (patients with the blood type A or AB).

**Fig. 3:**
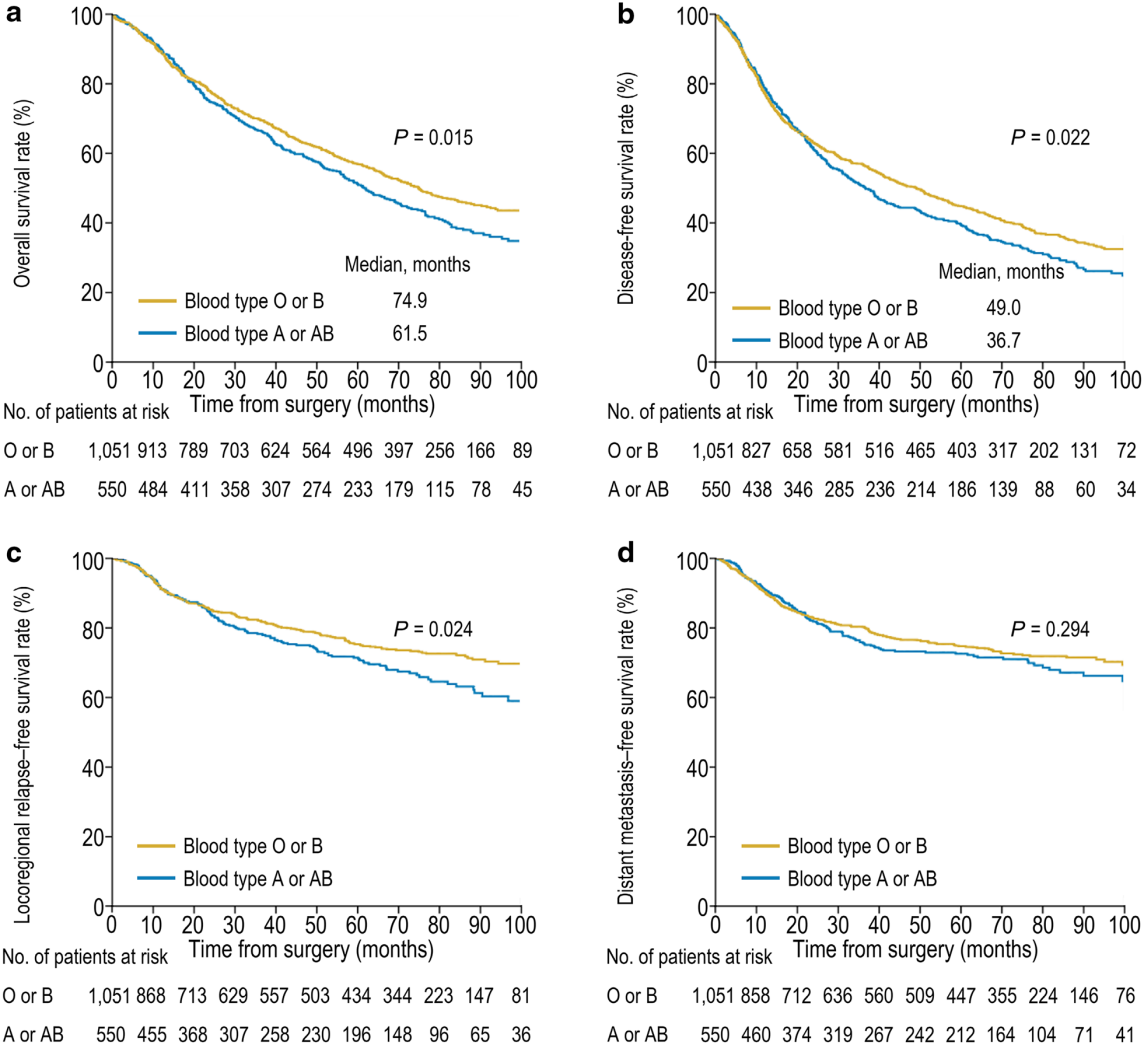
Kaplan–Meier survival curves for 1601 patients with curatively resected non-small cell lung cancer. **a** Group 1 (patients with the blood type O or B) had significantly prolonged overall survival compared with group 2 (patients with the blood type A or AB). **b** Group 1 had significantly prolonged disease-free survival compared with group 2. **c** Group 1 had significantly prolonged locoregional relapse-free survival compared with group 2. **d** No significant difference was observed for distant metastasis-free survival between groups 1 and 2

The OS, DFS, LRFS, and DMFS curves are shown in Fig. 3. Group 1 had significantly prolonged OS compared with group 2, with a median OS of 74.9 months compared with 61.5 months, respectively [hazard ratio (HR) 0.83; 95% CI 0.72–0.96; *P* = 0.015; Fig. 3a]. Additionally, group 1 had a significantly longer DFS (HR 0.86; 95% CI 0.76–0.98; *P* = 0.022; Fig. 3b) and LRFS (HR 0.79; 95% CI 0.64–0.98; *P* = 0.024; Fig. 3c) than group 2. However, no significant difference was observed for DMFS between group 1 and group 2 (HR 0.89; 95% CI 0.72–1.10; *P* = 0.294; Fig. 3d).

The association of OS with clinicopathologic characteristics was further analyzed using univariate and multivariate analyses. In the univariate analysis, males, smoking history, pT category of 3/4, pN category of 1/2, stage IIIA, pneumonectomy, no chemotherapy, and the blood type A/AB were identified as negative prognostic factors. When these variables were further analyzed in the multivariate analysis, we found that smoking status, pT category, pN category, pathologic tumor-node-metastasis (pTNM) stage, chemotherapy, and blood group had significant HRs, indicating that they were significant predictors of survival (Table 2).

**Table 2:** Univariate and multivariate analyses for overall survival (OS) of NSCLC patients

Variable	Median OS (months)	Univariate analysis		Multivariate analysis	
		HR (95% CI)	*P* value	HR (95% CI)	*P* value
Age					
≥55 years vs. <55 years	67.8 vs. 72.2	1.08 (0.93–1.25)	0.32	–	–
Gender					
Males vs. females	66.5 vs. 76.6	1.17 (1.01–1.37)	0.04	1.03 (0.82–1.28)	0.82
ECOG PS score					
1 vs. 0		0.97 (0.90–1.04)	0.36	–	–
Smoking status					
Ever vs. never	64.1 vs. 75.8	1.19 (1.03–1.37)	0.02	1.22 (1.06–1.40)	0.01
Pathology					
Squamous vs. non-squamous	71.4 vs. 69.1	1.00 (0.87–1.16)	0.98	–	–
pT category					
3/4 vs. 1/2	31.0 vs. 79.1	2.06 (1.76–2.41)	<0.01	1.61 (1.35–1.91)	<0.01
pN category					
≥N1 vs. N0	39.5 vs. NA	2.89 (2.51–3.33)	<0.01	2.42 (1.95–3.00)	<0.01
pTNM stage					
III vs. I–II	34.6 vs. NA	2.83 (2.46–3.25)	<0.01	1.44 (1.16–1.76)	0.01
Surgery type					
Pneumonectomy vs. others	28.2 vs. 72.5	1.80 (1.41–2.30)	<0.01	1.24 (0.96–1.60)	0.11
Chemotherapy					
No vs. yes	57.9 vs. 83.8	1.36 (1.19–1.57)	<0.01	1.31 (1.12–1.53)	<0.01
Blood type					
Group 2 vs. group 1	61.5 vs. 74.9	1.20 (1.04–1.38)	0.01	1.37 (1.10–1.70)	<0.01

*CI* confidence interval, *HR* hazard ratio, *NA* not available. Other abbreviations as in Table 1

## Discussion

Recently, the association between the ABO blood type and survival of cancer patients has drawn much attention. When this study was designed, the association between the ABO blood type and survival of patients with curatively resected NSCLC had not yet been explored. In this retrospective study of Chinese patients with completely resected NSCLC, patients with the blood type O or B had longer OS than those with the blood type A or AB. The blood type O or B was also associated with prolonged DFS and LRFS.

Our results of the association between the blood type O and patient survival are consistent with previous studies on other malignancies. The survival advantage of patients with the blood type O has been reported in pancreatic cancer [11, 12], breast cancer [13, 14], renal cell carcinoma [15], and nasopharyngeal carcinoma [16]. The blood type O appears to be a protective factor in the prevention of tumor development [21]. Our results of the association of the blood type A with patient survival are in close agreement with the results from previous studies. The blood type A has been reported to be associated with poor prognosis in patients with pancreatic cancer [11], nasopharyngeal carcinoma [16], and colon cancer [17]. However, controversy still exists about the relation between the ABO blood type and patient survival because many publications reported negative results [22–26]. As our study was completed, the first report concerning the association between the ABO blood type and survival of patients with resected NSCLC was published [27]. The results of that study showed that the ABO blood type was an independent prognostic factor for resected NSCLC, and the blood group A antigen might be associated with poor prognosis of patients with resected NSCLC. The number of patients was relatively small (*n* = 333), and some patients did not undergo complete resection in their study [27]. In our study, by contrast, the number of patients was relatively large (*n* = 1601), and all patients received curative resection for NSCLC.

Although the associations of the ABO blood type with cancer risk and survival have been reported in several malignancies [11–17], the genetic or biological mechanisms underlying the associations remain unclear. The ABO gene encodes three glycosyltransferases, which attach *N*-acetylgalactosamine, d-galactose, and no sugar residue to the H antigen backbone to form blood types A, B, and O, respectively [6]. In addition to their expression on the surface of red blood cells, ABO blood group antigens are expressed on the surface of cells from the gastrointestinal tract, urogenital tract, bronchopulmonary duct, skin, and breast duct [28, 29]. Loss of blood group antigen A/B expression on cancer cells is regulated by hypermethylation of the ABO gene promoter, which is an early event in tumor development [30]. Loss of ABO blood group antigens from tumor cells is associated with poor prognosis and increased metastatic potential in NSCLC [18, 31, 32]. Lee et al. [18] reported that the 28 patients with the blood type A or AB who had antigen A-negative tumors had significantly shorter survival than the 43 patients with the blood type A or AB who had antigen A-positive tumors and the 93 patients with the blood type O or B free of antigen A (median survival: 15 months versus 71 and 39 months, *P* < 0.001 and *P* = 0.002, respectively). These results may partially explain the positive association between the blood type A and poor prognosis in patients with NSCLC.

Other potential mechanisms underlying the association between the ABO blood types and patient survival include the host inflammatory state. Single nucleotide polymorphisms (SNPs) at the ABO locus have been reported to be associated with circulating levels of tumor necrosis factor-alpha (TNFα), soluble intracellular adhesion molecule-1 (sICAM-1), E-selectin, and P-selectin [33–36]. These serum molecules are associated with inflammatory responses that are associated with the processes of angiogenesis, tumor growth, invasion, and migration. Tumor development is induced by the inflammatory microenvironment, which consists of inflammatory cells and inflammatory mediators [37]. In particular, chronic inflammatory conditions predispose individuals to multiple types of malignancies and are linked to tumor invasion and metastasis [38]. The study by Suadicani et al. [39] suggested that the predictive values of inflammation-related risk factors for lung cancer mortality, including smoking history, high salt consumption, high alcohol intake, and occupational dust exposure, were high among males with the blood type O compared with those with the blood type A. Thus, the inflammatory state could be a possible mechanism explaining the association between the ABO blood types and patient prognosis.

This study nevertheless has several limitations that should be noted. The crucial disadvantage of this analysis is its retrospective nature. Because all patients were in-hospital, the possibility of selection bias cannot be ruled out. Another limitation is that East Asians constitute most of our study population. The monotonicity of the study population limits the universality of our results. Furthermore, only limited variables could be included in the multivariate analysis. Other factors, such as anaplastic lymphoma kinase (ALK) rearrangement status, may be significant prognostic factors for NSCLC, but these data are not currently available. Future well-designed studies that include diverse ethnic populations are warranted to further investigate the prognostic role of the ABO blood types in NSCLC patients. Additionally, other potential clinicopathologic factors should be considered.

## Conclusions

The ABO blood types were associated with survival of Chinese patients with curatively resected NSCLC. Patients with the blood type O or B had significantly longer OS, DFS, and LRFS than those with the blood type A or AB. Potential mechanisms underlying this association warrant further investigation.

## Abbreviations

NSCLC: non-small cell lung cancer
HR: hazard ratio
CI: confidence interval
OS: overall survival
DFS: disease-free survival
LRFS: locoregional relapse-free survival
DMFS: distant metastasis-free survival
TNM: tumor-node-metastasis
GWAS: genome-wide association study
ECOG: Eastern Cooperative Oncology Group
TNFα: tumor necrosis factor-alpha
sICAM-1: soluble intracellular adhesion molecule-1
SNP: single nucleotide polymorphism
ALK: anaplastic lymphoma kinase
